# Supplementary material for: Automated analysis of high‐content microscopy data with deep learning
Source: Mol Syst Biol. 2017 Apr 18;13(4):924. doi: 10.15252/msb.20177551 (PMC5408780; doi:10.15252/msb.20177551)
Supplement: Supplementary file 1 — Expanded View Figures PDF [file MSB-13-924-s001.pdf]

Expanded View Figures

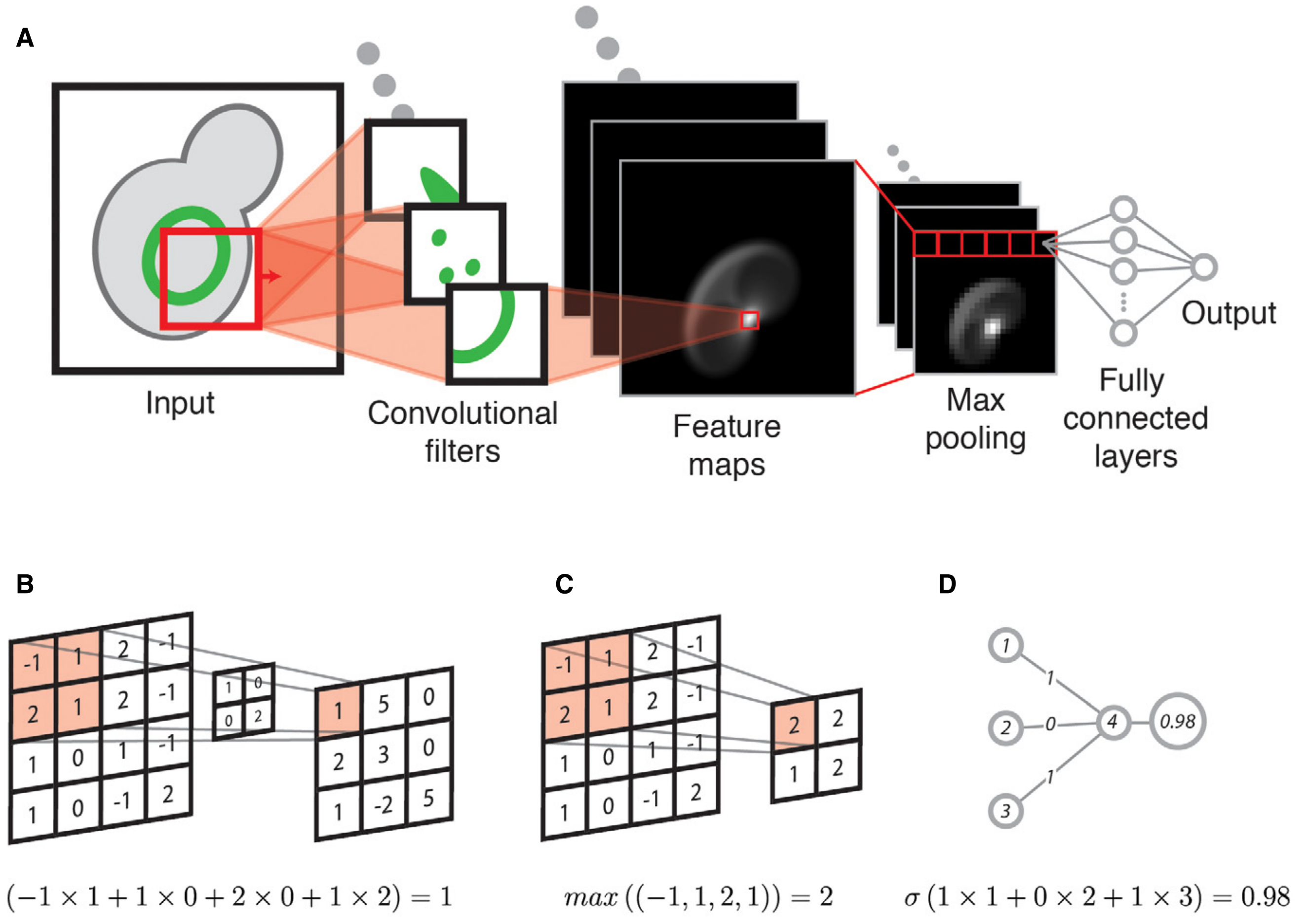

Figure EV1. Illustration of convolutional neural networks.

A Illustration of how convolutional neural networks learn to identify location invariant patterns. The input shown is an illustration of a yeast cell with a nuclear periphery protein localization. The input is convolved with convolutional filters, each representing a unique pattern that is learned during training. When the pattern of a filter matches the input at some location, the corresponding feature map is activated at that location. Pooling layers smooth the activations in the feature maps by calculating an aggregation (such as the maximum) over adjacent elements, effectively down sampling the feature maps. Pooling layers reduce the number of parameters in the model and also contribute to the location invariance of network. The fully connected layers in the network are typically responsible for classifying the activations extracted by the convolutional layers into the desired output categories. Each element in the final feature map is connected to each element in the first fully connected layer. The final activations in the network are passed through an activation function, such as the softmax function, to produce a distribution over output classes.

B An example of computation carried out by a convolutional filter. The calculations below the figure illustrate that the activation in top left corner is calculated by the weighted sum of its receptive field weighted by the convolutional filter. In convolutional networks, the values in the convolutional filters are parameters that are updated during training to reduce the networks prediction error on labeled samples from the training set.

C An example of computation carried out by max pooling layers. The calculations below the figure illustrate that the activation in the top left corner is the maximum over the elements in its receptive field. These layers do not have parameters and subsample the feature maps to reduce the number of parameters in the network and introduce more spatial invariance.

D An example of computation carried out by the fully connected layers. The calculations below the figure illustrate that the activation is the weighted sum of the input elements. Once again the weights themselves are the parameters learned by the network during training. A non-linear activation function is typically applied to this activation (as well as activations in other layers in the network). The non-linear activation functions enable the network to learn non-linear mappings between layers, and ultimately enable the network to approximate complex non-linear mappings between the input data and output classes. In the final layer, the sigmoid (σ) or softmax functions are used to produce distributions over the output classes for binary and multi-class problems, respectively.

 © 2017 The Authors

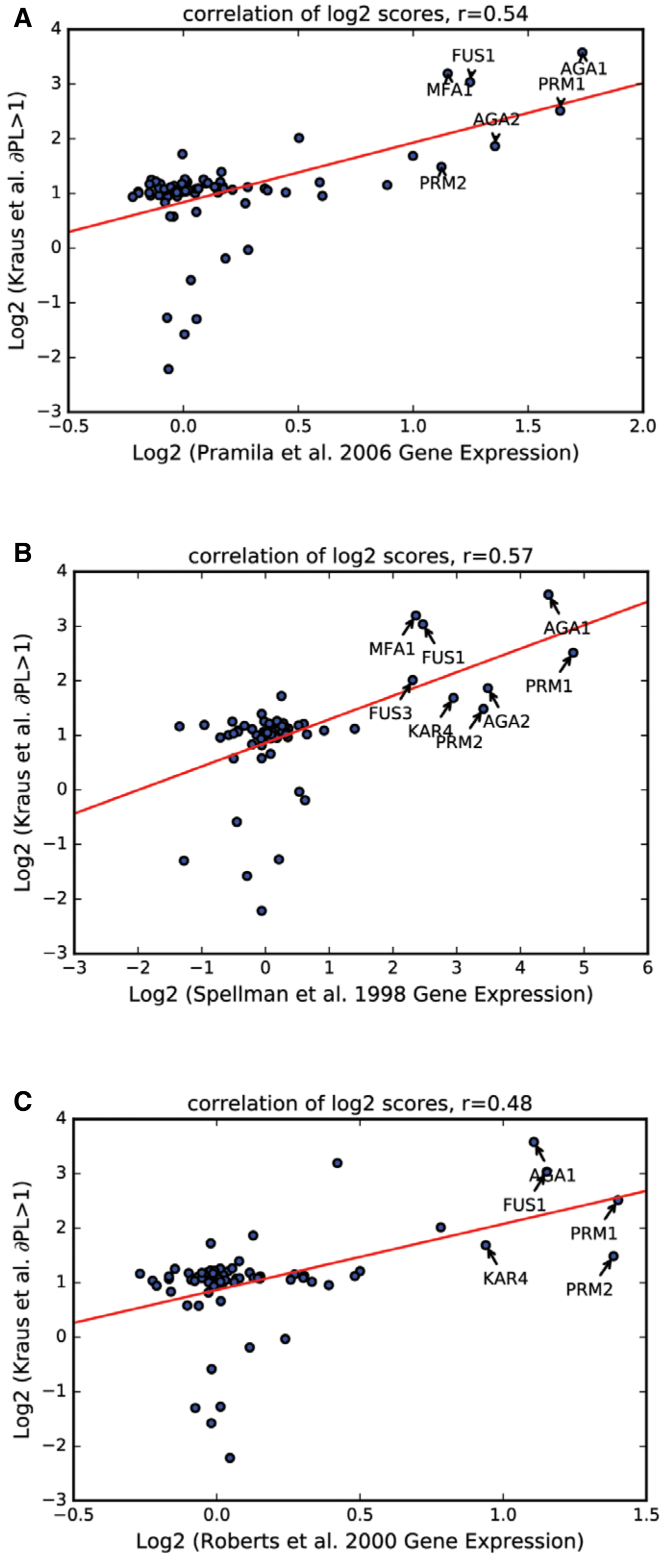

Figure EV2. Correlation of protein abundance measurements with gene expression data in response to α-factor treatment.

A–C In these plots, only proteins with $\partial PL > 1$ in at least one time-point are compared to corresponding gene expression changes from three different data sources. In each instance, genes demonstrating a substantial increase in expression as well as protein abundance are indicated on the plots. Comparison to gene expression microarray data from (A) Pramila *et al* (2006), (B) Spellman *et al* (1998), and (C) Roberts *et al* (2000).

© 2017 The Authors

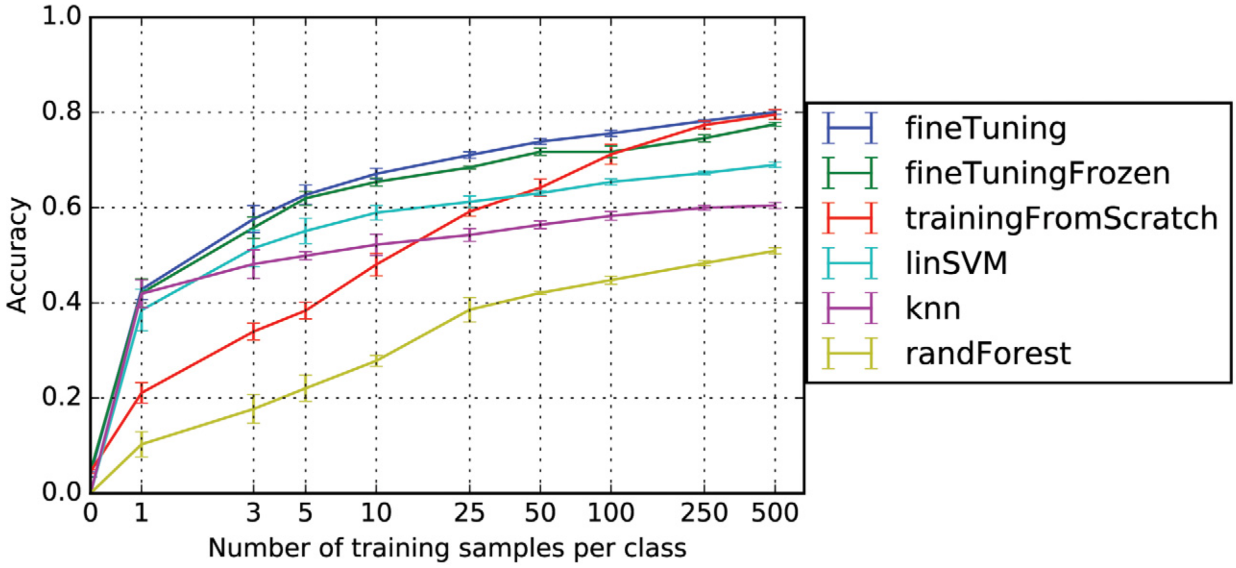

Figure EV3. Comparing methods for transferring DeepLoc to a new dataset generated in our group (WT-2017).

Comparison of classification accuracy (*y*-axis) for different training set sizes (*x*-axis) when training different classifiers on features extracted from DeepLoc. Most classifiers were implemented in Scikit-learn (Pedregosa *et al*, 2012). fineTuning—fine-tuning the entire network; fineTuningFrozen—fine-tuning only the fully connected layers (i.e., not updating the convolutional layers); trainingFromScratch—training the entire model from random initializations; linSVM—one-vs-one linear SVM trained on features extracted from the second last fully connected layer (fc2), (implementation: sklearn.multiclass.OneVsOneClassifier(sklearn.svm.SVC(kernel = "linear", C = 0.025)); knn—k-nearest neighbors trained on features extracted from the second last fully connected layer (fc2), (implementation: sklearn.neighbors.KNeighborsClassifier(n), n = 3 if more than 10 samples used per class, otherwise n = 1); randForest—random forest trained on features extracted from the second last fully connected layer (fc2), (implementation: sklearn.ensemble.RandomForestClassifier(n_estimators = 5)).

 © 2017 The Authors
