# Supplementary figures and images for: Automated analysis of high‐content microscopy data with deep learning
Source: Mol Syst Biol. 2017 Apr 18;13(4):924. doi: 10.15252/msb.20177551 (PMC5408780; doi:10.15252/msb.20177551)

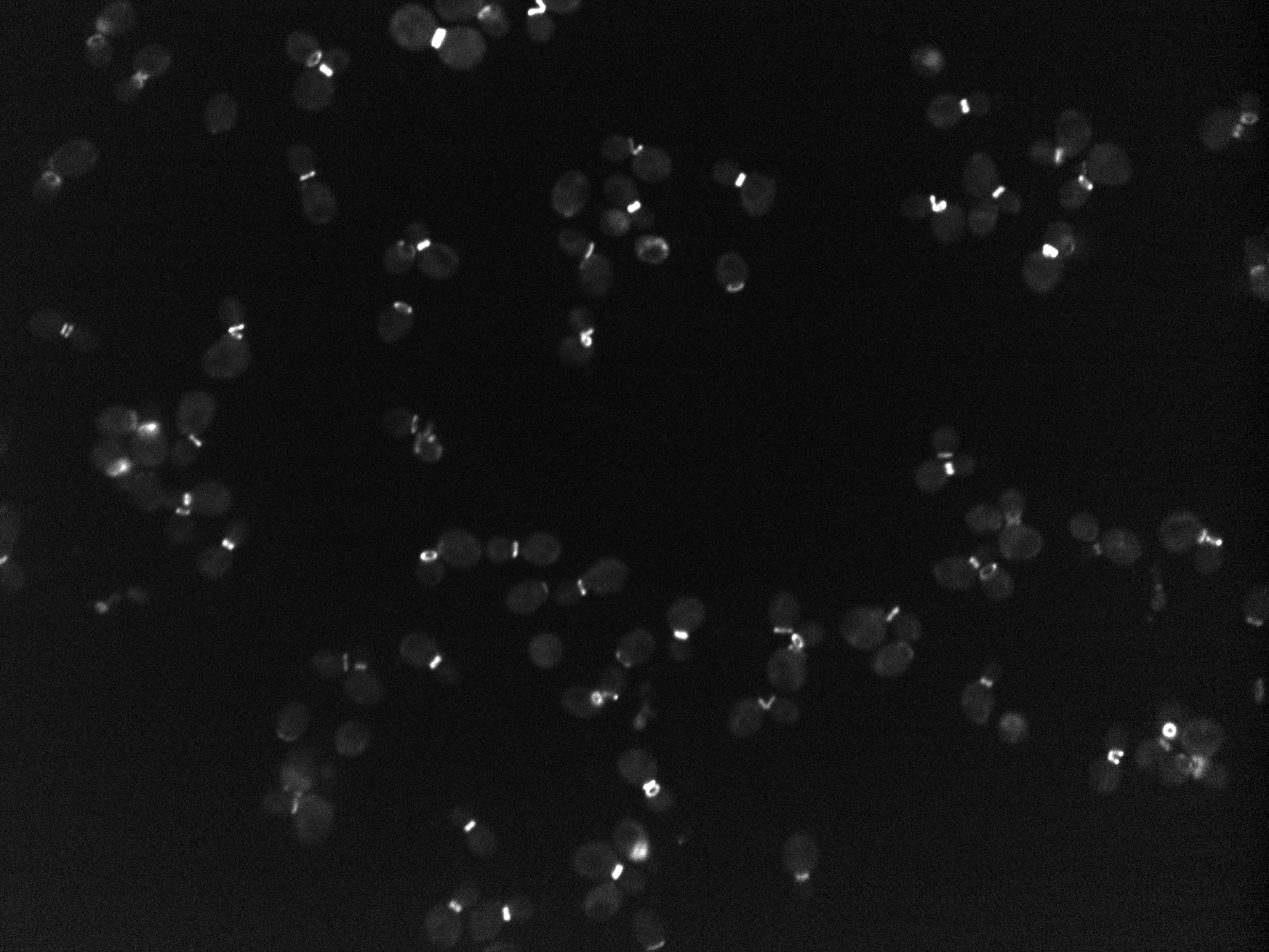

Supplement: Supplementary file 5 — Code EV1 [file MSB-13-924-s005.zip › Computer_Code_EV1/sample_image/plate01/007020000.flex]
